# Indents

**Published:** 1909-03-15

**Authors:** 


### INDENTS.

**☞ Brief Articles of Technical or Scientific Value will receive notice and comment. Contributions invited.**

*Arsenic Application.* In applying arsenic to a cavity that extends below the gum, moisten the cavity with eucalyptol, and completely fill with temporary stopping. Drill a hole through the stopping to the point where you wish to make the application, apply the arsenic, close the opening with more gutta-percha.—Dr. E. W. Tennant, in *Cosmos.*

*Removal of Amalgam Fillings.* Hold a heated instrument on the filling until the heat is felt in the tooth. Bur out at once, when it can be cut like cheese. Use an instrument having a very slender shank with a bulbous end, one of the old "Wood's Metal" fillers of forty years ago. The slender shank prevents the heat from radiating too rapidly.—A. H. Brockway, in *Cosmos.*

*The Rubber-dam.* The average dentist should have the dam applied to his own teeth by some careless man at least once a week. He would then use care in applying it for others. If in cold weather you would warm the clamp your patient would bless you. If in carrying the ligature between the teeth you are careful not to let it strike the gum suddenly, your patient will appreciate it.—F. M. Smith, in *Cosmos.*

*Root Canal Treatment.* More than one-half of the pulp canals treated become well in spite of he treatment and not from it. With regard to drilling out canals, personal experience leads to the thought that in certain cases, where it is possible and feasible, it is a wise procedure. In cases where it is not possible I rely upon thorough desiccation and flooding the canal with formol and vaporizing the formol with hot silver or copper wire. It is the vapor of formol that disinfects.—G. B. Hayes, *Dental Review.*

*Occlusion of Artificial Teeth Perfected by Carborundum.* When I had made the occlusion of an upper set of artificial teeth I am now wearing as accurate as I could with touch paper and the grind stone, I took carborundum into my mouth, and by grinding it between my teeth, I have produced an occlusion that is just as smooth and just as perfect as the occlusion of natural teeth.—Dr. G. V. Black, Chicago, *Dental Review,* Dec. 1908.

*Stability of Copper Alloys.* Has the test of time shown the modern amalgams to be any better than some of the older ones? Have you ever noticed that an amalgam filling which has become quite black upon the surface has not changed form to an appreciable extent? Is it because there is a large amount of copper in the alloy? The fact is, the flow of an amalgam under occlusion is a more serious factor than contraction among modern amalgams.—*Dominion Journal.*

*Removal of a Devitalized Pulp.* One of the simplest and most efficient methods of removing a devitalized or desensitized pulp is to wind a few fibres of cotton upon a smooth broach and pass it up on one side of a canal, with a gentle, rotating movement, and when as far into the canal as desired, rotate several times, which will entangle the fibres of cotton into the pulp and when the broach is withdrawn the pulp will be withdrawn also.—*Dominion Journal.*

*Cover for Instrument or Bracket Table.* I find the little paper napkins used in restaurants excellent for this purpose. They make a noiseless cover they are clean, are but little affected by moisture and can be quickly changed. Simply roll them up from each side, taking with them any scraps of gold or alloy, etc., to be taken care of later. They are inexpensive, costing about 10 cents per hundred, and can be had in a variety of sizes, patterns and colors. Use two or three at a time, one over the other, according to the thickness desired.—Dr. F. E. Cloud, New York City.

*To Soften Moldine Which Has Become Hard.* It often happens that from frequent use or long exposure to air, moldine loses plasticity. The usual method of adding glycerine is more or less unsatisfactory, even when all instructions are carefully followed. The best process to use is to place the moldine to be softened in a vessel, to which a sufficient quantity of water, to cover the moldine, is added. One or two spoonfuls of glycerine are added to the water. The moldine is then heated until the water has evaporated after which the moldine may be found having its original plasticity.—*Le Laboratoire.*

*Painless Puncturing of Alveolar Abscess* An alveolar abscess which is about to point may be opened without pain or the knowledge of the patient by dipping an explorer into carbolic acid and placing the end of the explorer against the mucous membrane at the point where the pus is nearest to the surface and gradually scratching it, as the tissue is cauterized by the carbolic. Soon a hole may be worked through to the cavity. The explorer will have to be dipped into the carbolic frequently to carry enough of the fluid to continue the cauterization and the anesthetization.—*Dominion Journal.*

*Donaldson Broach, a Suggestion.* Dr. J. H. Stephan suggests that before using a Donaldson broach for opening a pulp canal by the sulphuric acid method of Dr. Callahan, the extreme sharp point should be removed by means of an Arkansas stone. To test whether it is right or not, attempt to stick it into the thumb nail; if it slides off without scratching, it is right. Unless this is done the sharp point will stick into the side of the canal at the first curve or obstruction it meets, instead of passing around it, this produces a little spur at this point of the canal and prevents further progress, if indeed it does not wrongly direct the broach and lead to perforating the wall.—*Brief.*

*Formalin for Sensitive Cavities.* If a pellet of cotton be dipped in formalin (I use Schering's 40 percent. solution), passed through the flame of an alcohol lamp and placed in the cavity for a moment, a sensation of comfort is at once noticed by the patient. On removing the cotton and drying the cavity with warm air the excavation may be continued with noticeable less distress to the patient. In addition to its obtunding effect the repeated use of formalin by its disinfectant action renders less likely the recurrence of decay. I am satisfied that the obtunding effect is not due entirely to the heat and desiccation.—Dr. Geo. P. Marvin, Rochester, New York.

*To Refine Scrap.* A simple way to refine scrap (such as a dentist usually has—filling and clippings from gold plate, *not filings*) is to place it in a pit dug out of a piece of beechwood charcoal which is close grained and not very liable to check. For a flux use equal parts of borax and saltpetre, and sprinkle freely from time to time while the gold is kept *boiling* with the blowpipe until visually it shows refinement—clear and free from oxidation, and very fluid. It is now ready to put in the flask crucible and melted for casting. The longer the gold is "boiled" with the high-efficiency flame, the purer the gold will become; but there are some metals that in an alloy exert a persistent undesirable influence without going through the very best process of refinement known.—R. B. Fuller, in *American Journal.*

*An easy way to pack a Rubber Plate so That Colored Rubber will not Show Through the Pink.*

If you are to use two colors of rubber dissolve some of each color in chloroform using a separate bottle for each color. Have each solution about the consistency of thick cream. Heat the flask over a Bunsen flame until quite warm. With a small brush paint the plaster where you wish to use pink rubber with a pink solution, and with the other solution where you wish to use the colored rubber. Pack pink rubber where you painted the pink solution and pack the other rubber where you desire. When you pack in the sheet rubber against the wall where you have painted the solution, it will adhere to the coating and stay exactly where you place it. By judicious use of this method, almost any design, however fantastic, may be vulcanized in colored rubbers.—*Digest.*

*Cast Cap and Dowel forBadly Broken-down Roots.*

This method consists of taken impression of enlarged root canal and broken end of the root, using a piece of iridio platinum wire gauge 20, and length of dowel to carry the wax to end of canal. The impression was then carved up leaving a cap over end of root and square post to receive the crown and whole thing cast in pure gold with the iridio platinum wire in the center.

A matrix of platinum 1..1000 was made as follows: A tube was made approximately the size of the square post which was soldered to a plate of platinum of same thickness. This then was burnished over post and cap.

A Davis crown was then used to form the crown by cutting out a section mesio distally approximating the size of square post. This was attached to matrix while in position on cap by means of beeswax and crown and matrix removed. The crown and matrix were held in position by hawk bill pliers and beeswax removed and Consolidated porcelain body used to fill in space between matrix and crown and then fused, thus completing the crown with the same body throughout.—C. J. Lyons, in *Summary*.

*Future Dentistry.* If there is one thing that appeals to me it is that there has been developed within the last few years the fact that dentistry has a future such as never before has been conceived. I can see conditions for the better that I never saw before. I am an optimist, and I believe that an optimist in dentistry has a red and rosy apple, while the pessimist will quarrel over the core. Some say that "an optimist is one who doesn't care what happens, provided it doesn't happen to him," but I am not that kind of a man, and I believe there never was so much in the future of dentistry as there is now. But it must come along the lines of organization by societies taking up the idea of scientific investigation and taking money from their treasuries and giving it to people who show ability along certain lines.

In my opinion the dentist of the future will be a man who takes all beneficial things in all the professions, into dentistry, and uses them. That is the future that I see, nor do I believe that we will fail because intelligent agitation when fought for by any organization of any great strength will accomplish wonders. Intelligent agitation will do almost anything, and I trust the time will come when all organizations will have these thoughts and these ideas concerning the advancement of dentistry come to them—come welling and surging in:—Dr. A. Flanagan, in *Items of Interest*.

*About Vulcanite Repairs.* Dovetailing is utterly useless. All that is necessary is to have a clean, new surface, and the new and old rubber will "weld."

The fact is that if the case is heated as hot in repairing as it was in vulcanizing, the first time, the old plate liquefies and the new and old run together.

If the old plate only semi-liquefies (under less heat than the first time), then the new liquid rubber is still driven into the old softened plate, when the two still weld. The requisite is a clean, new surface on the old plate.

But there is too much repairing done. If, in the first place, a plate is vulcanized at as low temperature as possible, and long enough to harden it only sufficiently for a finish, and is then left in the flask for several hours, not only to cool but to "season," it will be difficult, if possible, to break it at all.

But in most cases it is just about as easy and simple and requires little more time, to renew or reproduce an entire plate from the old broken one as to patch it. A sagacious dentist can easily explain, with good and honest reasons, and get extra pay for a new plate.

It is no trouble to make a rubber plate that *will not break*, provided you use good rubber, vulcanize properly, between metal surfaces and do not let the material come in contact with plaster. Use mercurial-tin paste to dissolve the tin foil off the plate.—J. W. Greene, in *Western Journal.*

*A Good Inlay in fifteen minutes.* A gold inlay can be made in fifteen minutes in those cases where no contour is required. For example, a lower right second molar, occlusal cavity, involving the central fossa and the four grooves radiating from it. Prepare the cavity so an impression will draw, not being paritcular to remove all the decay from the bottom of the cavity before the impression is taken. The decay may be removed after the inlay is made, and will then provide for retention of the cement in the great majority of cases. Take the impression in a hard, sticky wax held in a small tray. Fill the impression with Price's artificial stone, and immediately place it upside down on a piece of asbestos over a Bunsen flame. As the wax is melted out the stone hardens. A couple of minutes later apply a full blow-pipe flame on the stone, which completes its hardening. Burnish either pure gold or thin platinum into the cavity, and into this pack crystal gold, not too tightly, and then fuse 22K. plate into the crystal gold. It will soak it up as a sponge does water if the stone is well heated from below. The model, inlay, and all may be cooled in water without destroying the model. Now proceed to finish the inlay down to the margins with stones, and polish at the lathe. Split the model with cutting pliers, and the inlay will fall out ready to set. If there is any fear of fusing a pure gold matrix, platinum may be used, and gold and platinum crystals packed into the cavity, and pure gold fused into it. Do not read this over and forget it, but do the first case you meet in the manner described, and then you will always remember it.—*Dominion Dental Journal.*

*New Moulds for Artificial Teeth.* There is much that is excellent in the agitation for new moulds of artificial teeth, especially in the occlusal surfaces of bicuspids and molars.

This agitation, however, overlooks one important consideration. It is,that very few of us make intelligent selections among the moulds we now have. Such unscientific selections are not the fault of the manufacturer;they lie wholly with the dentist, and they are directly traceable to lack of definite knowledge on the part of the dentist. Until the average of knowledge in this particular line is increased, new moulds, however perfect, will be of but little value.

What is the first consideration in selecting artificial teeth? Is it not size?

Yet how many dentists can tell just the size of the teeth indicated for a given full upper? How many dentists know what steps to take to find out the required sizes? How many of those dentists who are most intelligent and painstaking select teeth by trying them on the model? Yet this is a crude method and full of shortcomings. Teeth for full dentures can be selected intelligently only from the bite; not from the model.

We need new moulds—true, but we need first and more to learn to select teeth intelligently. We shall then find that many of the present moulds are better than we thought. And when the new moulds come, we shall be ready to use them intelligently.

But unless we learn to select teeth with discretion, the new moulds may also fail at our hands. And the fault will not be with the moulds; it will be with *us*.—Dr. Clapp, in *Digest*.

*The Eye Sight.* After the forty-fifth year of age all eyes undergo a change, and the sight for reading and for sewing begins to fail. For the distance the sight may be as clear as ever, but for things near by it is poor. For this reason many people can tell the time by the clock, but not by the watch. When a person in the forties with seemingly good sight becomes sleepy while reading and complain that the light is poor and the newspaper print is bad, and at the same time holds the paper at arm's length, glasses are needed. To read without them is foolish and injurious.

The two serious eye affections that we should always look out for when a person of advanced years complains that the sight is failing quicker than it should are cataract and glaucoma. In the cataract, the lens of the eye loses its clearness and prevents the light from entering the eye. There is no pain and it takes from one to ten years before the sight is completely shut off. An operation affords the only relief. In glaucoma there is considerable pain, caused by pressure inside the eye. The eye is hard and has a staring appearance, and the sight rapidly fails, unless the trouble is speedily relieved by an operation.

The selection of glasses, especially for astigmatism and eye-strain, requires great care, and should be entrusted to a physician only. To try on different glasses at an optician's store is like sampling the different bottles in a drug store for the one that will do the most good. Most people who choose their own glasses, select a pair that is too strong and therefore unsuited to the eyes. No one should ever get so-called pebbles. They are no better than good glass, and people who pay for pebbles seldom get them.

People who use glasses to work during the day should be provided with another and a stronger pair for the night.

We hear a great deal about the harmfulness of artificial light. Good artificial light of any kind is far better than poor daylight. Another popular fallacy concerns the use of a veil, which many consider harmful to the eyes. A veil protects the eyes, not only from the dust and wind, but also from the bright light. It often takes the place of dark glasses and rests the eyes.—D. A. Bardes in *Dietic and Hygienic Gazette.*

*Prevention of Cancer.* Not less than sixty-five to seventy-five per cent. of all cancers in women occur in atrophied organs.

As regards the influence of chronic inflammations and irritations, much can be done, and here is our most hopeful field for prevention. Warts and birthmarks that are in any way subject to pressure or friction from clothing or movements should be promptly removed, as both show a distinctly greater tendency than normal tissue to develop into cancer. Cracks, fissures, chafes and ulcers of all sorts, especially about the lips, tongue, mammary gland, uterus and rectum, should be early and aseptically dealt with. Jagged remnants of teeth should be removed, all suppurative processes of the gums antiseptically treated, and the whole mouth-parts kept in a thoroughly aseptic condition.

Thorough and conscientious attention to this sort of surgical toilet work is not only valuable for its preventive effect—which is considerable—but also because it will insure the bringing under competent observation at the earliest possible moment of the beginnings of true cancer.

For the disease itself, after it has once started, there is, like treason in the body politic, but one remedy—capital punishment. Parleying with the rebels is worse than useless. Pastes, caustics, X-rays, trypsin, radium—all are fatally defective, because they suppress a symptom only and leave the cause untouched. Only in one form of surface cancer, the so-called flat-celled or rodent ulcer, which has little or no tendency to form spore cells and attack the deeper organs, are they effective.

Nothing is easier and nothing more idle than to destroy and break down cells which have actually become cancerous; but so long as there remains in the body a single nest, or even cell, of the organ in which the revolt started, so long the life of the patient is in danger.

Absolutely the only remedy which is of any value is complete removal with the knife. The one superiority of the knife, shudder as we may at the name of it, over every other means of removal lies solely in this fact, that with it can be removed not merely the actual cancer, but the entire gland or group of surrounding cells in which this malignant, parricidal change has begun to occur.

The modern radical operations for cancer take not merely the tumor, but the entire diseased breast, for instance, and all the lymph glands into which it drains, clear up to the armpit, with the muscles beneath it down to the ribs. When this is done early enough the disease does not recur. Such radical and complete amputation of an organ or region as this is possible in from two-thirds to three-fourths of all cases if seen reasonably early.

With watchfulness and courage, our attitude toward the cancer problem is one of hopeful confidence.—Dr. Woods Hutchinson, in *Saturday Evening Post.*

